# Impact of in ovo and water supplementation of a postbiotic on intestinal integrity and immune responses in broiler chickens challenged with necrotic enteritis

**DOI:** 10.3389/fvets.2025.1654028

**Published:** 2025-08-13

**Authors:** Bingqi Dong, Ali Calik, Rami A. Dalloul

**Affiliations:** ^1^Avian Immunobiology Laboratory, Department of Poultry Science, University of Georgia, Athens, GA, United States; ^2^Department of Animal Nutrition & Nutritional Diseases, Faculty of Veterinary Medicine, Ankara University, Ankara, Türkiye

**Keywords:** postbiotic, necrotic enteritis, stem cell, immunity, in ovo

## Abstract

Necrotic enteritis (NE) is an enterotoxemic disease of poultry caused by *Clostridium perfringens* and inflicts substantial economic losses in the poultry industry. Postbiotics have emerged as a promising mitigation approach for NE as they can improve birds’ performance and nutrient absorption and reduce NE-associated pathology. This study evaluated the effects of in ovo and post-hatch application of a postbiotic on intestinal health and the response of broilers during a subclinical NE challenge. On embryonic day (d) 18, fertile broiler eggs were injected with 0.2 mL of either water or a postbiotic into the amnion. Male hatchlings (*n* = 288) were randomly assigned to one of four groups (six replicate cages, 12 birds/cage): (1) NC (in ovo water, no challenge), (2) PIW (postbiotic in ovo and in drinking water, no challenge), (3) NC+ (NC with NE challenge), and (4) PIW+ (PIW with NE challenge). On d 14, all birds in the NE-challenged groups were orally gavaged with 3,000 *E. maxima* sporulated oocysts, followed by two doses of ~1 × 10^8^ CFU/mL per bird of *C. perfringens* on d 19 and d 20. Intestinal permeability, jejunum and ileum histomorphology, and mRNA abundance of genes related to gut health and immune response in the jejunum, ileum, and cecal tonsils (CT) were assessed. Data were analyzed using Student’s *t*-test and two-way ANOVA, with significance set at a *p*-value ≤ 0.05. On d 14, the PIW birds exhibited reduced crypt depth (CD) and an increased villus height-to-crypt depth (VH: CD) ratio in the jejunum. In addition, mRNA abundance of mucin-2 and olfactomedin-4 was higher in the PIW birds, while the levels of tumor necrosis factor alpha (TNF-α), inducible nitric oxide synthase (iNOS), interferon gamma (IFN-γ), and interleukin-10 were lower compared to the NC group. On d 21, intestinal permeability was not significantly affected, while postbiotic supplementation resulted in better villi and crypt structures, as manifested by a higher VH: CD ratio. Furthermore, the NE-challenged birds with postbiotic supplementation had higher mRNA abundance of zonula occludens-1 (ZO-1) and TNF-α in the jejunum and iNOS in the CT compared to the NE-challenged control group. In conclusion, supplementation of a postbiotic in ovo and via drinking water demonstrates potential to improve intestinal health and regulate immune responses during a subclinical NE challenge.

## 1 Introduction

Necrotic enteritis (NE) and coccidiosis are two of the most important diseases in the commercial poultry industry, causing significant economic losses on a global scale (1, 2). Coccidiosis, caused by *Eimeria* spp., is considered a major predisposing factor for the development of NE. Coccidial infection triggers a mucogenic response in the host, characterized by increased mucus production and altered intestinal microbiota, which creates an environment that favors the growth of *Clostridium perfringens* and promotes the onset of NE (3). Consequently, NE causes intestinal inflammation, disrupts the intestinal epithelial layer through necrosis, and alters the intestinal structure, the function of tight junction (TJ) proteins, and the immune response of broiler chickens.

Postbiotics are inanimate bacterial or microbial fermentation components beneficial to the host (4). *Saccharomyces cerevisiae*-derived yeast fermentation products are commonly used as postbiotics, containing functional metabolites produced through controlled and proprietary fermentation processes. Postbiotics consist of a complex profile of metabolites, including short-chain fatty acids (SCFAs), organic acids, antioxidants, cell wall polysaccharides (such as β-glucan and mannan), amino acids, nucleotides, and vitamins B and K (5). These components have been associated with a wide range of beneficial outcomes across species, including dairy cattle (6), swine (7), poultry (8, 9), and horses (10). Supplementation of a postbiotic in ovo and in drinking water promoted gut health by reducing NE lesion scores and enhancing the capacity of nutrient digestion and absorption during an NE challenge in broiler chickens (11). Further, postbiotic supplementation in drinking water improved gut barrier function and intestinal morphology by increasing villus height (VH) and the villus height-to-crypt depth (VH: CD) ratio, reducing inflammatory responses, and modulating plasma phosphorus levels in heat-stressed ducks (12).

The effects of postbiotics on intestinal barrier function and the immune response have been studied previously. Pekin ducks supplemented with *S. cerevisiae*-derived yeast fermentation products exhibited higher mRNA abundance of intestinal TJ proteins, including claudin, occludin (OCLN), zonula occludens-1 (ZO-1), and mucin-2 (MUC2), compared to birds that were fed a basal diet (13). In addition, dietary supplementation with postbiotics increased CD3+, CD4+, and CD8 + T-lymphocyte populations in the spleen and blood, along with increased IgM levels in fresh peripheral blood, suggesting an improvement in both cellular and humoral immunity in broilers challenged with *E. tenella* (14). Similarly, cell wall components (a combination of β-glucan and mannoproteins) from *S. cerevisiae* positively influenced gut signaling during the NE challenge by promoting cell growth and reducing inflammation in broiler chickens (15).

In ovo administration of dietary nutrients into broiler eggs can enhance the development of the embryo, promote early gut maturation, boost the development of embryonic gut microbiota, and stimulate the maturation of post-hatch gut-associated lymphoid tissue (16, 17). However, few studies have evaluated the effects of early postbiotic supplementation on intestinal integrity and immune response during subclinical NE. Therefore, this study investigated the combined effects of postbiotic supplementation in ovo and in drinking water post-hatch on intestinal permeability, intestinal morphology, and mRNA abundance of structural and immune response genes in broilers during a subclinical NE challenge.

## 2 Materials and methods

### 2.1 Incubation and in ovo application

This study was approved by and conducted according to the guidelines of the UGA Institutional Animal Care and Use Committee (AUP #A2023 02–001). On embryonic day 18 (E18), fertilized Ross 708 eggs were candled to remove any unfertilized or dead embryos and randomly assigned to two groups. Each egg was candled, the air cell outlined, and a pinhole was made using a sterile 18-gauge needle attached to a rubber stopper. According to the manufacturer’s recommendations, 1.6 mL of an *S. cerevisiae*-based liquid postbiotic was diluted in one liter of sterile water. A second 1-mL syringe with a 22-gauge needle was used to inject 0.2 mL of either sterile water or the diluted postbiotic (1.6 mL/L) into the amnion. After injection, the pinhole was sealed with sterile paraffin wax, and the eggs were returned to the incubator at 37.5°C with 65% humidity.

### 2.2 Treatment, management, and NE challenge

Upon hatch, the chicks were feather-sexed, and 288 male hatchlings were housed in battery cages with six replicates, each containing 12 chicks, for 21 days. The treatment groups were as follows: (1) NC (in ovo water injection, no challenge), (2) PIW (postbiotic in ovo and in drinking water, no challenge), (3) NC+ (NC with NE challenge), and (4) PIW+ (PIW with NE challenge). The chicks were reared in battery cages equipped with trough feeders and waterers. Waterers were cleaned daily, and 1.6 mL/L of a fresh postbiotic/water mixture was provided to the PIW and PIW+ treatment groups throughout the experimental period. A corn-soybean meal diet was formulated for the starter (d 0–14) and grower (d 15–21) phases to meet the nutrient requirements of the Ross 708 broilers. Feed and water were provided ad libitum. On d 14 post-hatch, all birds in the NE-challenged groups (NC+ and PIW+) were orally gavaged with 3,000 *E. maxima* sporulated oocysts per bird. *C*. *perfringens* (#CP6, *netB* positive) was cultured anaerobically overnight at 37°C in fluid thioglycolate medium, and on d 19 and d 20, the birds were orally gavaged with 1 mL of fresh culture containing approximately 1.0 × 10^8^ CFUs per bird.

### 2.3 Intestinal permeability

A fluorescein isothiocyanate-dextran (FITC-d; MW 4000 Da; Sigma Aldrich, Canada) solution was freshly prepared on each sampling day, stored at 4°C, and wrapped in aluminum foil to avoid light exposure. On d 14 and d 21, one bird per cage was randomly selected, leg banded, and inoculated with the FITC-d solution (2.2 mg/mL/bird) via oral gavage (18). After 2 h of inoculation, the birds were euthanized, and blood was collected from the jugular vein into pre-labeled sterile vacutainers. The blood samples were kept in a dark container at room temperature for 3 h and centrifuged at 3,000 × *g* for 15 min to separate the serum. A standard solution was made by diluting FITC-d with pooled sera from six non-experimental control birds. The serum samples and standard solution (100 μL each) were transferred to a 96-well dark flat-bottom plate in triplicate wells, and fluorescence was measured using a microplate reader (BioTek, Winooski, VT, United States) at 485 nm excitation and 528 nm emission.

### 2.4 Intestinal histomorphology

On d 14 and d 21, sections of the midpoint of the jejunum and ileum from one bird per cage were collected, rinsed with phosphate-buffered saline (PBS), fixed in 10% neutral formalin for at least 24 h, and embedded in paraffin. Sections of 5 μm thickness were cut and stained with hematoxylin and eosin (H&E). The H&E-stained sections were imaged using a Keyence microscope (BZ-X810; Keyence, Osaka, Japan) at 40 × magnification. Brightfield microscopy images were used to measure intestinal histomorphology parameters, including VH and crypt depth (CD), using ImageJ (National Institutes of Health, Bethesda, MD, United States). A minimum of 12 villi and crypts from four independent sections were measured per bird. VH was measured from the villus-crypt junction to the tip of the villus. CD was measured from the base of the crypt to the top of the crypt.

### 2.5 RNA extraction and real-time PCR

On d 0, 12 birds per treatment group were randomly selected and euthanized by cervical dislocation. Subsequently, one bird per cage was randomly selected on d 7, d 14, and d 21 for sampling. Approximately 2–3 cm of tissue samples were collected from the mid-jejunum and mid-ileum, and cecal tonsil (CT) tissues were excised, snap-frozen in liquid nitrogen, and stored at −80°C for mRNA abundance of genes related to intestinal integrity, stem cell proliferation, and immune response. The tissue samples were homogenized using a Bead Mill Homogenizer (VWR International, Radnor, PA, United States), and the total RNA was extracted using TRIzol reagent following the manufacturer’s instructions (Invitrogen Life Technologies, Waltham, MA, United States). The total RNA concentration was measured by optical density (OD) at 260 nm (Nanodrop One^C^, Thermo Fisher), and purity was assessed by evaluating the OD 260/280 ratio. After extraction, 4 μg of the total RNA was reverse-transcribed into cDNA using the qScript cDNA SuperMix (Quantabio) as per the manufacturer’s protocol. The relative mRNA abundance of intestinal integrity genes [claudin (CLDN) 1 and 3, OCLN, ZO-1, and MUC2], stem cell-related genes [leucine-rich repeat-containing G-protein-coupled receptor 5 (LGR5) and olfactomedin-4 (OLFM4)], and immune response-related genes [tumor necrosis factor alpha (TNF-α), inducible nitric oxide synthase (iNOS), interferon gamma (IFN-γ), interleukin-1 beta (IL-1β), and IL-10] were measured using PowerTrack™ Fast SYBR Green Master Mix (Applied Biosystems, Waltham, MA, United States) on a QuantStudio™ 3 Real-Time PCR System (Applied Biosystems). To confirm amplicon specificity, each reaction included a melting curve analysis. The RT-PCR primers are listed in Table 1. Relative mRNA abundance of target genes was calculated using the 2^-ΔΔCt^ method (19), and glyceraldehyde 3-phosphate dehydrogenase (GAPDH) was used as the housekeeping gene. The calibrator for all target genes was the average ΔCT value of the non-challenged control group (NC) at each respective time point.

**Table 1:** Sequences of the primers used for the analysis of mRNA abundance via quantitative RT-PCR.

Gene^1^	Primer Sequence^2^	Size	GenBank no.	Reference
CLDN1	F: GTGTTCAGAGGCATCAGGTATC	107	NM_001013611.2	(45)
	R: TCAGGTCAAACAGAGGTACAA			
CLDN3	F: CCCGTCCCGTTGTTGTTTTG	126	NM_204202.1	(45)
	R: CCCCTTCAACCTTCCCGAAA			
OCLN	F: CCGTAACCCCGAGTTGGAT	214	NM_205128.1	(45)
	R: ATTGAGGCGGTCGTTGATG			
ZO-1	F: GGAGTACGAGCAGTCAACATAC	101	XM_413773	(45)
	R: GAGGCGCACGATCTTCATAA			
MUC2	F: TTCATGATGCCTGCTCTTGTG	93	XM_421035	(45)
	R: CCTGAGCCTTGGTACATTCTTGT			
LGR5	F: CCTTTATCAGCCCAGAAGTGA	338	XM_425441.4	(46)
	R: TGGAACAAATGCTACGGATG			
OLFM4	F: GACTGGCTCTCTGGATGACC	108	NM_001040463.1	(46)
	R: AGCGTTGTGGCTATCACTTG			
TNF-α	F: CGTTTGGGAGTGGGCTTTAA	61	NM_204267.1	(47)
	R: GCTGATGGCAGAGGCAGAA			
iNOS	F: GGCAGCAGCGTCTCTATGACTTG	185	NM_204961	(48)
	R: GACTTTAGGCTGCCCAGGTTG			
IFN-γ	F: GCTCCCGATGAACGACTTGA	63	NM_205149.2	(45)
	R: TGTAAGATGCTGAAGAGTTCATTCG			
IL-1β	F: CGAGGAGCAGGGACTTTGC	71	NM_204524.2	(45)
	R: GAAGGTGACGGGCTCAAAAA			
IL-10	F: CGCTGTCACCGCTTCTTCA	63	NM_001004414.2	(47)
	R: CGTCTCCTTGATCTGCTTGATG			
GAPDH	F: CCTAGGATACACAGAGGACCAGGTT	64	NM_204305.1	(11)
	R: GGTGGAGGAATGGCTGTCA			

1 CLDN1, claudin 1; CLDN3, claudin 3; OCLN, occludin; ZO-1, zonula occludens-1; MUC2, mucin 2; LGR5, leucine rich repeat containing G protein-coupled receptor 5; OLFM4, Olfactomedin-4; TNF-α, Tumor necrosis factor alpha; iNOS, Inducible nitric oxide; IFN-γ, interferon gamma; IL-1β, interleukin 1 beta; IL-10, interleukin 10; GAPDH, Glyceraldehyde 3-phosphate dehydrogenase.

2 Reference chicken gene sequences for forward (F) and reverse (R; 5′-3′) primers, and the amplicon size (bp) and GenBank accession numbers used for primer design are listed.

### 2.6 Statistical analyses

Statistical analysis for the pre-challenge period (d 0–14) was performed using Student’s *t*-test. For the post-challenge period (d 14–21), data were analyzed as a 2 × 2 factorial design, with additive treatment (no postbiotic vs. postbiotic in ovo and in drinking water) and NE challenge (no challenge vs. NE challenge) as main effects, including their interaction. Analysis was performed using two-way ANOVA in JMP Pro 18, followed by Tukey’s test to compare means. All figures were created using the GraphPad Prism 8 software (San Diego, CA, United States). The results are presented as least square means (LS Means) ± standard error mean (SEM). Statistical significance between the groups was defined as a *p*-value ≤ 0.05.

## 3 Results

### 3.1 Intestinal permeability

**Figure 1:**
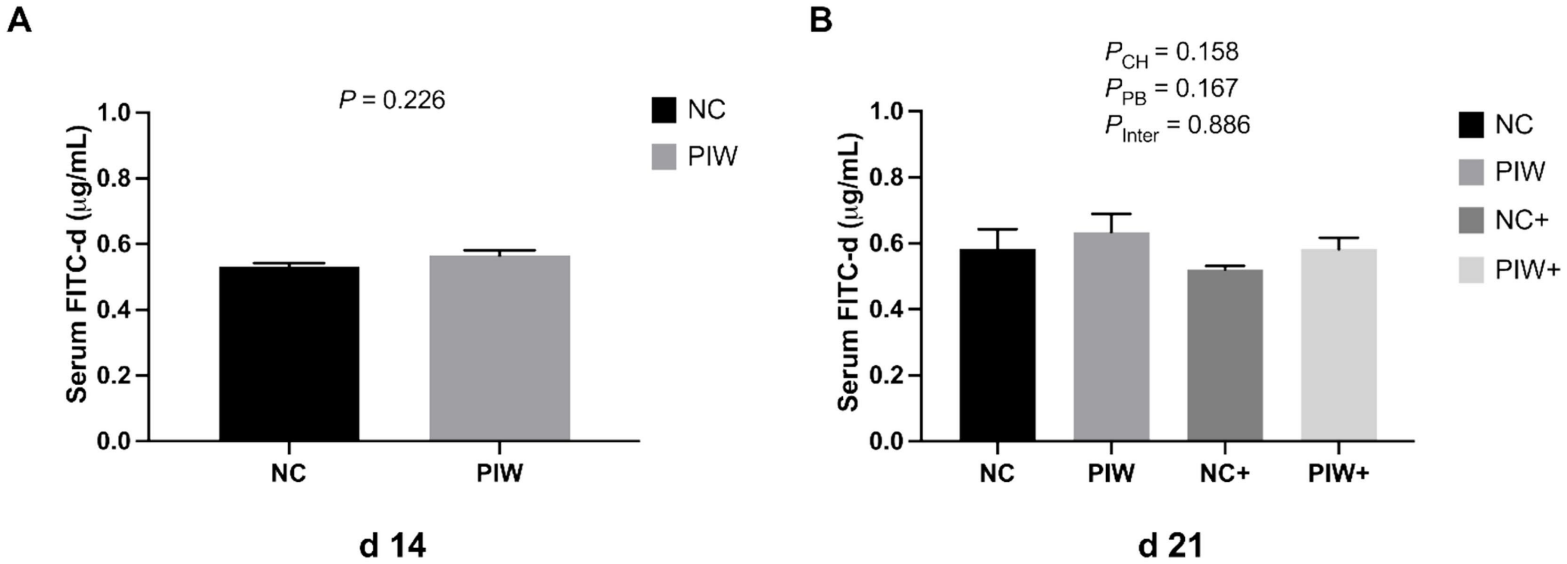
Serum fluorescein isothiocyanate-dextran (FITC-d, 4 kDa) levels on d 14 **(A)** and d 21 **(B)**. Data represent the LS means ± SE value. Panel **A** includes 12 birds per treatment, analyzed using Student’s *t*-test. Panel **B** includes six birds per cage, analyzed using two-way ANOVA. NC, in ovo water injection, drinking water, no challenge control group; PIW, in ovo postbiotic, postbiotic in drinking water, no challenge; NC+, in ovo water injection, drinking water, NE challenge; PIW+, in ovo postbiotic, postbiotic in drinking water, NE challenge.

Serum FITC-d levels in the broilers on d 14 and d 21 are presented in Figure 1. On d 14, serum FITC-d concentrations were not affected by in ovo or water supplementation of the postbiotic. In addition, no significant interaction of postbiotic treatment × NE challenge was observed, with the concentration of serum FITC-d unchanged at the peak of the NE challenge on d 21.

### 3.2 Histomorphological measurements

The morphological measurements of the jejunum and ileum on d 14 and d 21 are shown in Table 2. On d 14, significantly lower CD (*p* < 0.001) and a higher VH: CD ratio (*p* = 0.001) were observed in the jejunum of the PIW birds. In the ileum, no significant differences in VH, CD, or VH: CD ratio were observed on d 14.

**Table 2:** Effect of in ovo administration and water supplementation of the postbiotic on intestinal morphology during the NE challenge^1^.

Treatment groups^2^	Jejunum			Ileum		
	Villus height (μm)	Crypt depth (μm)	VH: CD	Villus height (μm)	Crypt depth (μm)	VH: CD
d 14						
NC	1105.2	150.3^a^	7.8^b^	618.1	133.8	5.1
PIW	1065.2	120.9^b^	9.4^a^	631.5	144.6	4.6
SEM	23.83	6.20	0.42	13.90	8.35	0.25
*P*-value	0.097	<0.001	0.001	0.364	0.198	0.066
d 21						
NC	1182.8^a^	146.6	8.4	665.5^c^	153.9^b^	4.5^b^
PIW	1119.2^a^	127.5	9.4	746.8^b^	112.0^c^	7.1^a^
NC+	698.3^c^	367.3	2.1	839.4^a^	217.8^a^	4.1^b^
PIW+	876.9^b^	355.3	2.7	821.9^a^	204.6^a^	4.2^b^
SEM	14.00	4.26	0.12	7.35	2.41	0.09
Main effects						
PB						
Water	940.5^b^	256.9	5.3^b^	752.4^b^	185.9^a^	4.3^b^
Postbiotic	998.1^a^	241.4	6.1^a^	784.4^a^	158.3^b^	5.6^a^
CH						
Non-challenge	1150.9^a^	137.0^b^	8.9^a^	706.1^b^	132.9^b^	5.8^a^
NE challenge	787.6^b^	361.3^a^	2.4^b^	830.7^a^	211.2^a^	4.1^b^
*p*-value^3^						
PB	0.041	0.070	0.001	0.031	<0.001	<0.001
CH	<0.001	<0.001	<0.001	<0.001	<0.001	<0.001
Inter	<0.001	0.675	0.435	0.001	0.003	<0.001

^a–c^Superscripts within columns indicate differences at *p* ≤ 0.05.

1 Values are expressed as LS means ± SE.

2 NC, in ovo water injection, drinking water, no challenge; PIW, in ovo postbiotic, postbiotic in drinking water, no challenge; NC+, in ovo water injection, drinking water, NE challenge; PIW+, in ovo postbiotic, postbiotic in drinking water, NE challenge.

3 PB, postbiotic treatment effect; CH, NE challenge effect; Inter, postbiotic treatment and NE challenge interaction.

On d 21, a significant postbiotic treatment × NE challenge interaction (*p* < 0.001) was observed for jejunal VH, where the NC+ and PIW+ groups exhibited significantly lower VH compared to the birds in the NC and PIW groups. Among the non-challenged birds, VH did not differ significantly between the NC (1182.8 μm) and PIW (1119.2 μm) groups. However, among the NE-challenged birds, VH was significantly higher in the PIW+ group (876.9 μm) compared to the NC+ group (698.3 μm). No significant interaction between postbiotic supplementation and NE challenge was observed for jejunal CD or the VH: CD ratio. The NE challenge significantly increased CD compared to the non-challenged birds (*p* < 0.001). The VH: CD ratio in the NE-challenged birds was significantly lower compared to the non-challenged birds (*p* < 0.001). In addition, postbiotic supplementation significantly increased the VH: CD ratio (*p* = 0.001), with higher values observed in the postbiotic groups compared to the non-supplemented groups.

There was a significant postbiotic treatment × NE challenge interaction effect on VH (*p* = 0.001), CD (*p* = 0.003), and the VH: CD ratio (*p* < 0.001) in the ileum on d 21. Specifically, VH and CD were significantly greater in the NC+ and PIW+ groups compared to the NC and PIW groups. The birds in the NC+ (839.4 μm) and PIW+ (821.9 μm) groups had significantly higher VH compared to both the NC (665.5 μm) and PIW (746.8 μm) groups. While there was no significant difference in VH between the NC+ and PIW+ birds, among the non-challenged groups, the PIW birds exhibited significantly higher VH compared to the NC birds. In addition, a significant postbiotic treatment × NE challenge interaction was observed for the VH: CD ratio (*p* < 0.001), where the PIW birds exhibited a significantly higher VH: CD ratio compared to the NC, NC+, and PIW+ groups in the ileum on d 21.

**Figure 2:**
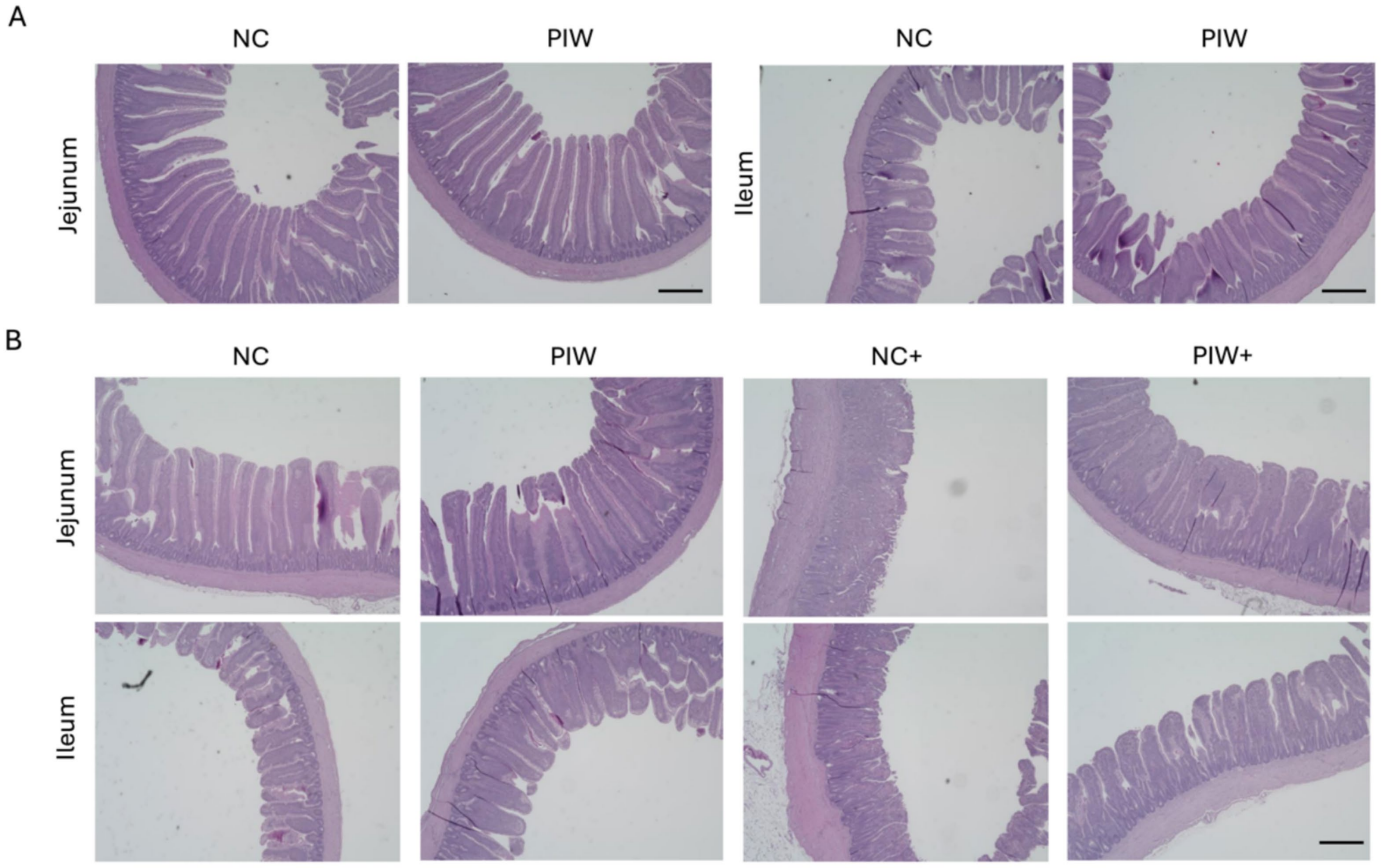
Histomorphological images of the jejunum and ileum on d 14 **(A)** and d 21 **(B)**. NC, in ovo water injection, drinking water, no challenge control group; PIW, in ovo postbiotic, postbiotic in drinking water, no challenge; NC+, in ovo water injection, drinking water, NE challenge; PIW+, in ovo postbiotic, postbiotic in drinking water, NE challenge. Scale bar: 500 μm.

Representative light microscopy images of intestinal morphology are shown in Figure 2. Severe damage to the jejunum was observed in the NC+ group, characterized by villus shortening, crypt hyperplasia, and fusion of villi and crypts. Postbiotic supplementation significantly alleviated the degree of intestinal epithelial damage (Figure 2B). Similarly, the NE-challenged birds with postbiotic supplementation (PIW+) showed improved ileal morphology compared to the NC+ group, although the severity of ileal damage was less pronounced than in the jejunum.

### 3.3 mRNA abundance of intestinal integrity genes in the jejunum and ileum on days 0, 7, and 14

**Figure 3:**
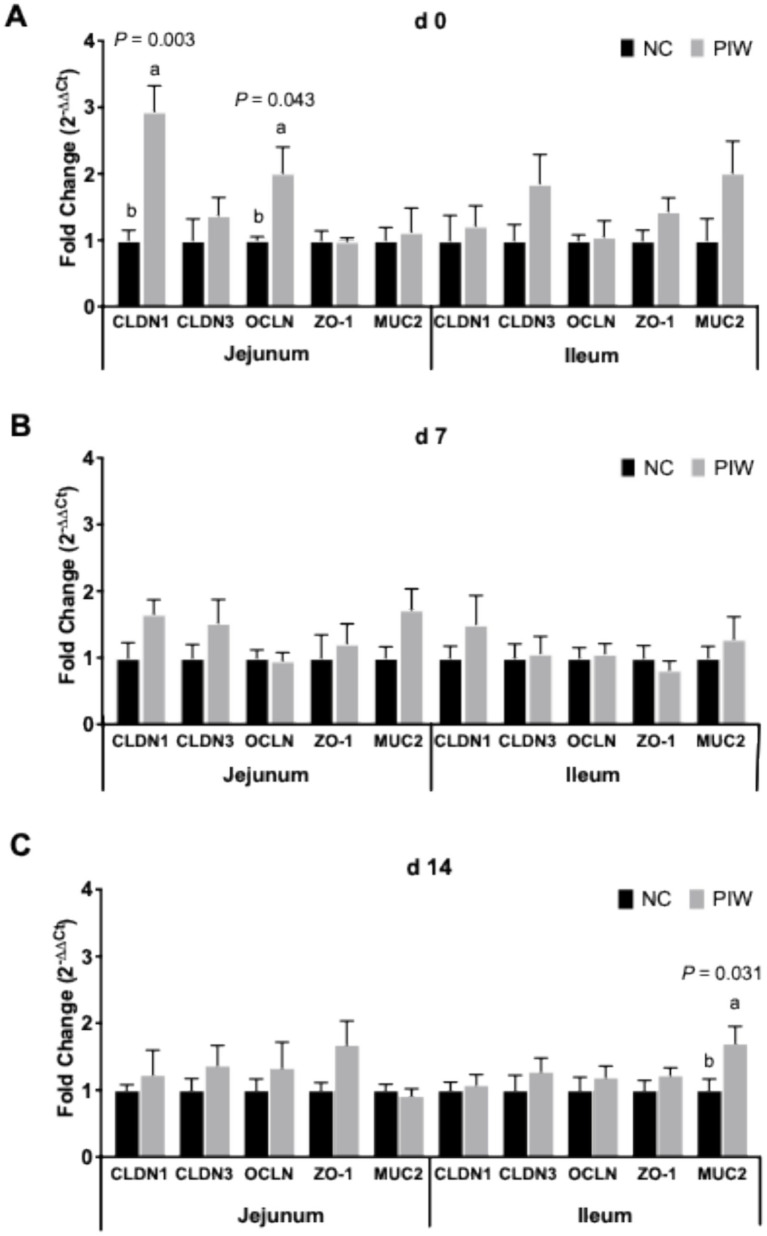
Relative mRNA abundance of intestinal integrity genes in the jejunum and ileum on d 0 **(A)**, d 7 **(B)**, and d 14 **(C)**. Bars with different letters (a,b) are significantly different (*p* ≤ 0.05). Data represent the LS means ± SE value of 12 birds per treatment. NC, in ovo water injection, drinking water, no challenge control group; PIW, in ovo postbiotic, postbiotic in drinking water, no challenge.

The effects of in ovo and water supplementation of the postbiotic on the mRNA abundance of intestinal integrity genes in the jejunum and ileum during the pre-challenge period are shown in Figure 3. mRNA abundance of CLDN1 (*p* = 0.003) and OCLN (*p* = 0.043) in the jejunum was higher in the postbiotic birds (PIW) compared to those in the NC group on d 0 (Figure 3A). However, no differences were observed between the PIW and NC birds for these intestinal integrity genes in the ileum on d 0, in both the jejunum and ileum on d 7, or in the jejunum on d 14 (Figures 3B,C). mRNA abundance of MUC2 (*p* = 0.031) was significantly higher in the PIW birds compared to the NC birds in the ileum on d 14 (Figure 3C).

### 3.4 mRNA abundance of intestinal integrity genes in the jejunum and ileum on d 21

**Figure 4:**
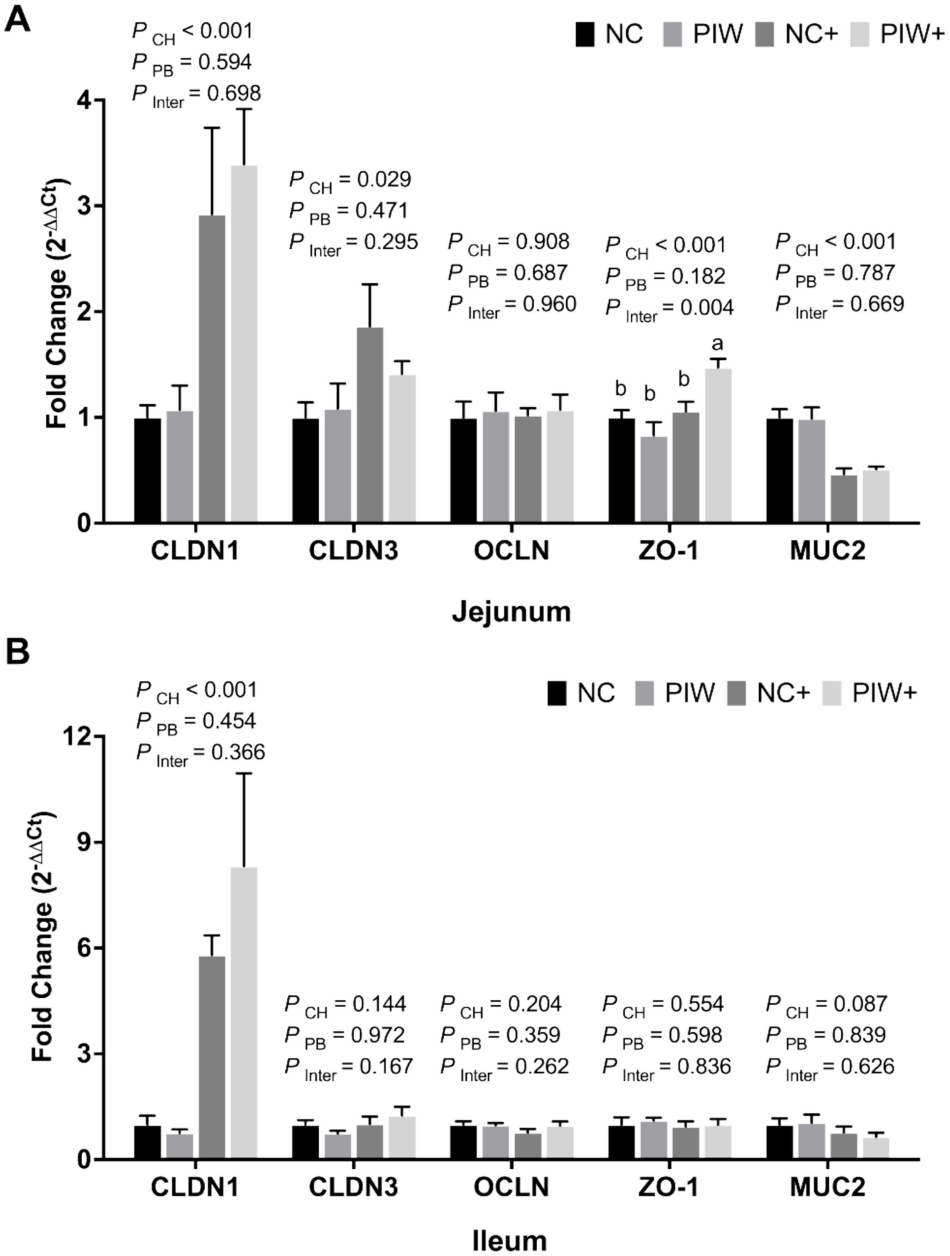
Relative mRNA abundance of intestinal integrity genes in the jejunum **(A)** and ileum **(B)** on d 21. Bars with different letters (a,b) are significantly different (*p* ≤ 0.05). Data represent the LS means ± SE value of six birds per cage. NC, in ovo water injection, drinking water, no challenge control group; PIW, in ovo postbiotic, postbiotic in drinking water, no challenge; NC+, in ovo water injection, drinking water, NE challenge; PIW+, in ovo postbiotic, postbiotic in drinking water, NE challenge.

A significant postbiotic and NE challenge interaction was observed between the treatment groups in the mRNA abundance of ZO-1 (*p* = 0.004) in jejunal tissues (Figure 4A), where the PIW+ treatment exhibited significantly higher ZO-1 mRNA abundance compared to the other treatment groups. In addition, a significant NE challenge effect was observed, with both challenged groups showing greater mRNA levels of CLDN1 (*p* < 0.001), CLDN3 (*p* = 0.029), and ZO-1 (*p* < 0.001) and lower MUC2 (*p* < 0.001) compared to the non-challenged groups in the jejunum on d 21 (Figure 4A). Similarly, a significant NE challenge effect was observed, with both challenged groups showing greater mRNA levels of CLDN1 (*p* < 0.001) compared to the non-challenged groups in the ileum on d 21 (Figure 4B).

### 3.5 mRNA abundance of stem cell markers in the jejunum and ileum on days 0, 7, and 14

**Figure 5:**
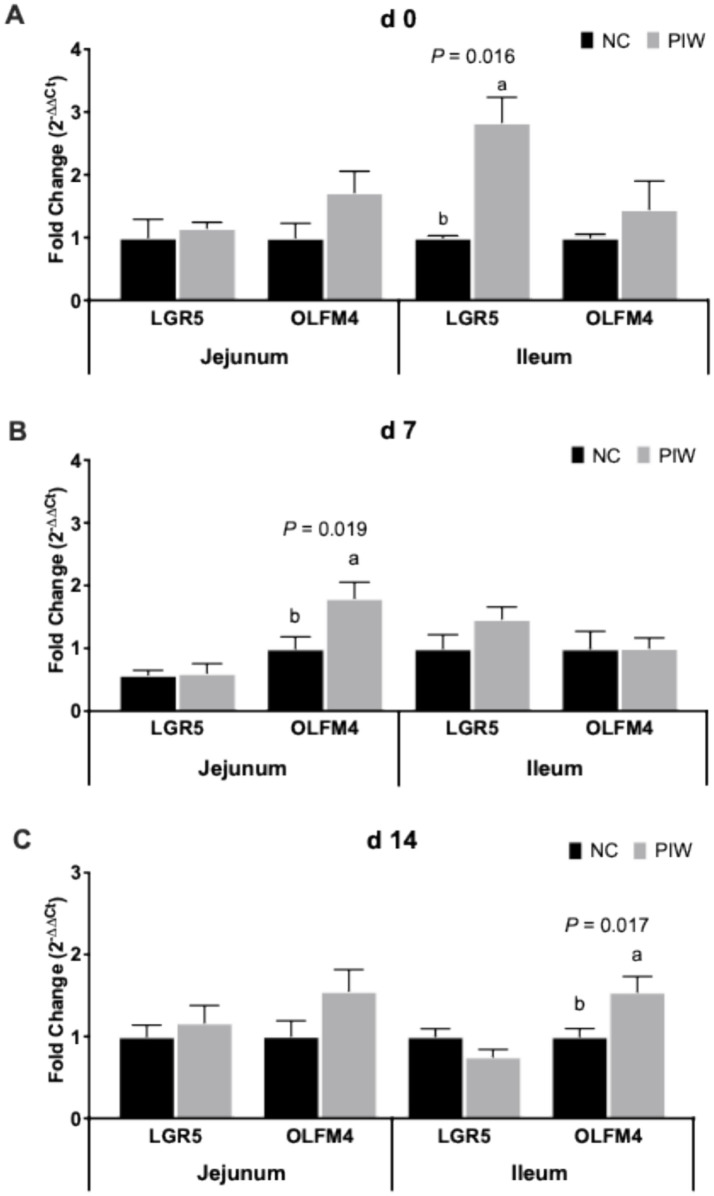
Relative mRNA abundance of stem cell markers in the jejunum and ileum on d 0 **(A)**, d 7 **(B)**, and d 14 **(C)**. Bars with different letters (a,b) are significantly different (*p* ≤ 0.05). Data represent the LS means ± SE value of 12 birds per treatment. NC, in ovo water injection, drinking water, no challenge control group; PIW, in ovo postbiotic, postbiotic in drinking water, no challenge.

The effects of in ovo injection and water supplementation of the postbiotic on the mRNA abundance of stem cell markers in the jejunum and ileum of the broilers are shown in Figure 5. On d 0, no significant differences were found for stem cell markers in the jejunum, but mRNA abundance of LGR5 was significantly higher (*p* = 0.016) in the PIW group compared to the NC group in the ileum. A significantly greater mRNA abundance of OLFM4 (*p* = 0.019) was observed in the PIW group compared to the NC group in the jejunum on d 7, and on d 14, OLFM4 levels were significantly higher (*p* = 0.017) in the PIW group compared to the NC group in the ileum.

### 3.6 mRNA abundance of stem cell markers in the jejunum and ileum on day 21

**Figure 6:**
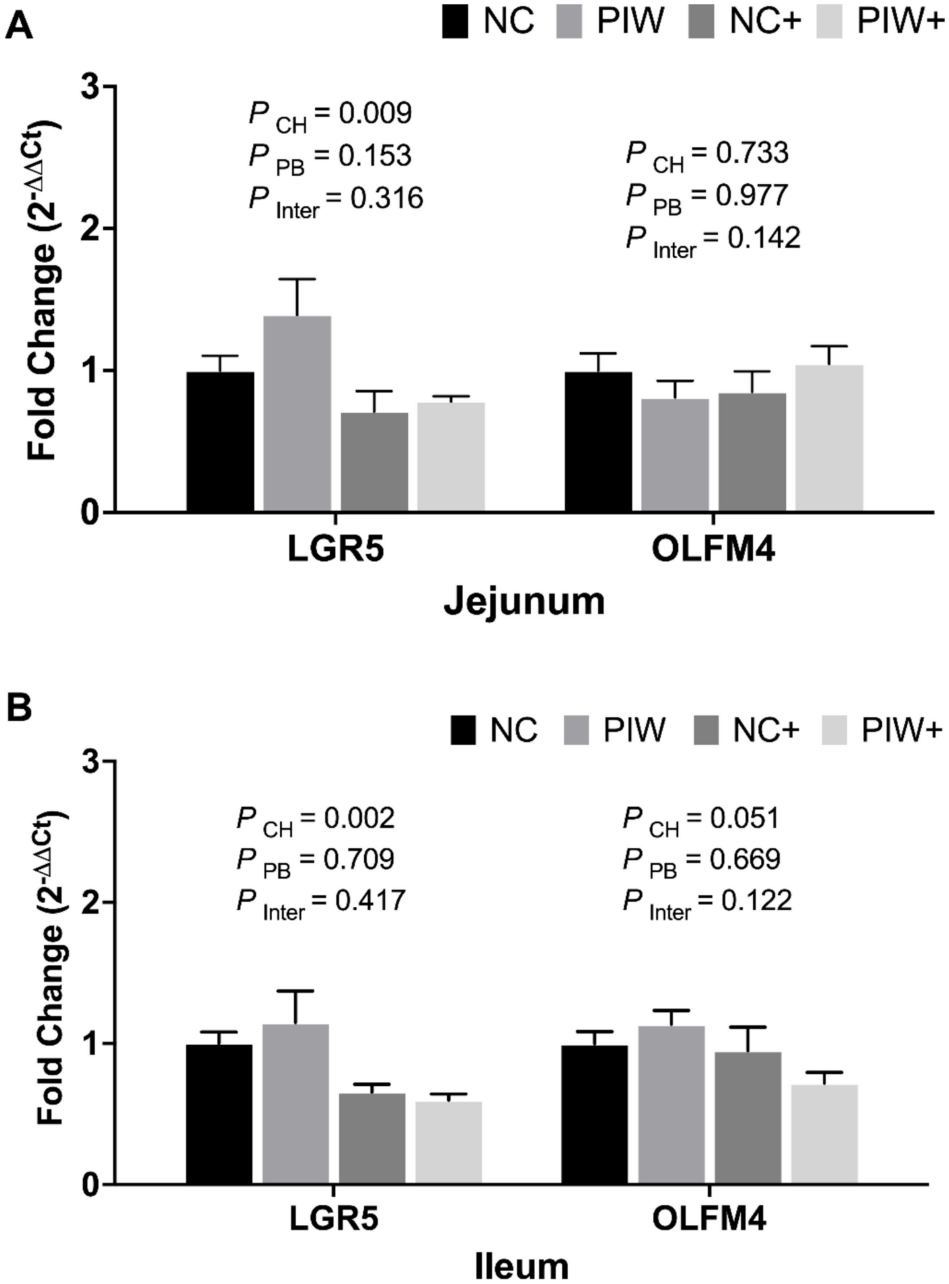
Relative mRNA abundance of stem cell markers in the jejunum **(A)** and ileum **(B)** on d 21. Data represent the LS means ± SE value of six birds per cage. NC, in ovo water injection, drinking water, no challenge control group; PIW, in ovo postbiotic, postbiotic in drinking water, no challenge; NC+, in ovo water injection, drinking water, NE challenge; PIW+, in ovo postbiotic, postbiotic in drinking water, NE challenge.

On d 21, no significant postbiotic treatment × NE challenge interactions were observed for stem cell markers in both the jejunum and ileum (Figure 6). However, the NE challenge resulted in significantly lower mRNA levels of LGR5 in the jejunum (*p* = 0.009) and ileum (*p* = 0.002) on d 21. The postbiotic treatment had no significant impact on the stem cell markers analyzed in the jejunum and ileum on d 21.

### 3.7 mRNA abundance of immune response genes in the jejunum and CT on days 0, 7, and 14

**Figure 7:**
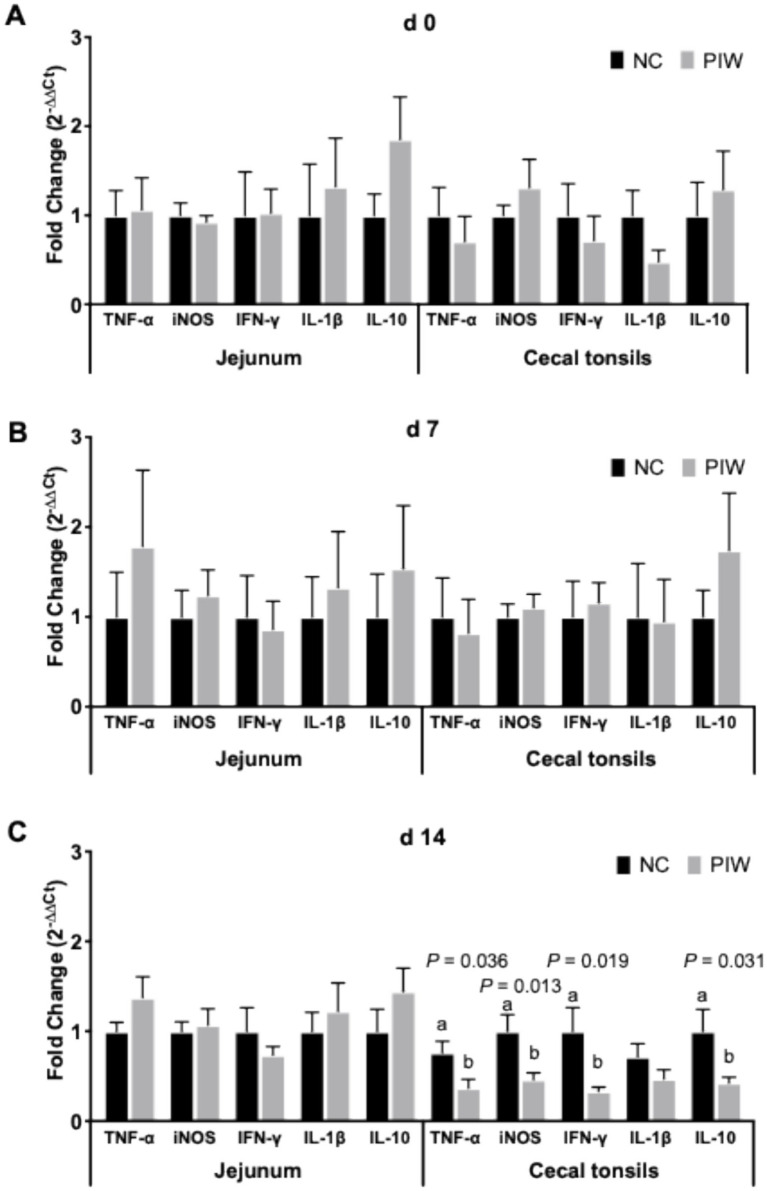
Relative mRNA abundance of immune response genes in the jejunum and cecal tonsils on d 0 **(A)**, d 7 **(B)**, and d 14 **(C)**. Bars with different letters (a,b) are significantly different (*p* ≤ 0.05). Data represent the LS means ± SE value of 12 birds per treatment. NC, in ovo water injection, drinking water, no challenge control group; PIW, in ovo postbiotic, postbiotic in drinking water, no challenge.

The effects of in ovo and post-hatch water supplementation of the postbiotic on the mRNA abundance of immune response genes in the jejunum and CT of the broilers are shown in Figure 7. There was no significant difference in the mRNA abundance of immune-related genes in either the jejunum or CT on d 0 and d 7 (Figures 7A,B). On d 14, no significant differences were found for the immune response genes in the jejunum. However, mRNA abundance of TNF-α (*p* = 0.036), iNOS (*p* = 0.013), IFN-γ (*p* = 0.019), and IL-10 (*p* = 0.031) was lower in the PIW group compared to the NC group in the CT on d 14.

### 3.8 mRNA abundance of immune response genes in the jejunum and CT on d 21

**Figure 8:**
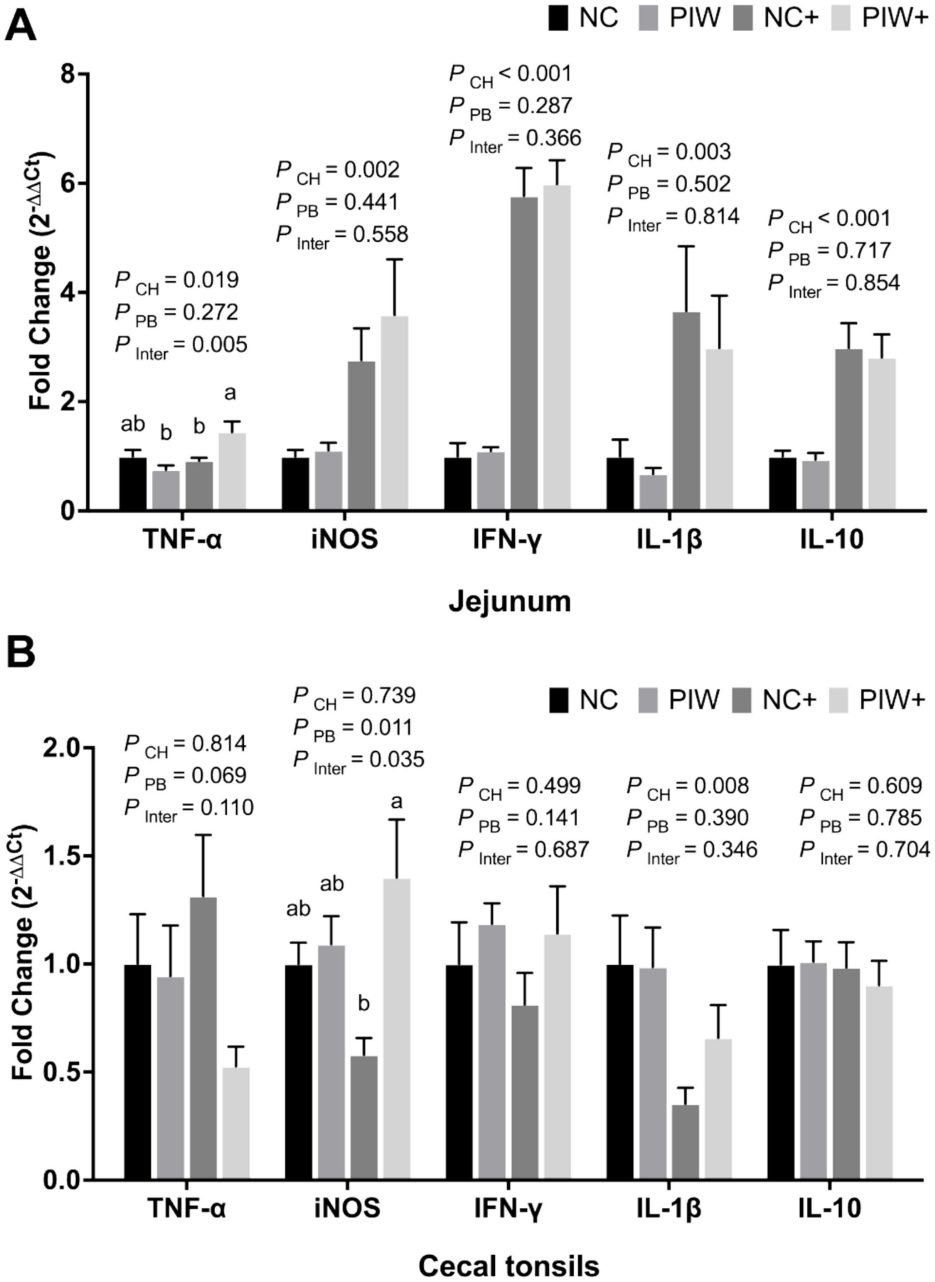
Relative mRNA abundance of immune response genes in the jejunum **(A)** and cecal tonsils **(B)** on d 21. Bars with different letters (a,b) are significantly different (*p* ≤ 0.05). Data represent the LS means ± SE value of six birds per cage. NC, in ovo water injection, drinking water, no challenge control group; PIW, in ovo postbiotic, postbiotic in drinking water, no challenge; NC+, in ovo water injection, drinking water, NE challenge; PIW+, in ovo postbiotic, postbiotic in drinking water, NE challenge.

A significant postbiotic treatment and NE challenge interaction was observed between the treatment groups for the mRNA abundance of TNF-α and iNOS in the jejunum and CT, respectively (Figures 8A,B). The NE challenge resulted in significantly lower mRNA abundance of TNF-α (*p* = 0.019), iNOS (*p* = 0.002), IFN-γ (*p* < 0.001), IL-1β (*p* = 0.003), and IL-10 (*p* < 0.001) levels in the jejunum, as well as reduced IL-1β (*p* = 0.008) in the CT on d 21. The postbiotic treatment had no significant impact on immune response genes in the jejunum but resulted in increased iNOS (*p* = 0.011) mRNA abundance in the CT on d 21.

## 4 Discussion

The NE challenge primarily causes tissue damage in the small intestine, leading to intestinal inflammation and compromising immune function. Subclinical NE is characterized by poor growth, mild intestinal lesions, and no to minimal NE-related mortality (20). We previously reported that in ovo and water supplementation of a postbiotic alleviated the negative effects of an NE challenge by improving performance, reducing NE lesions, and increasing the mRNA abundance of intestinal nutrient transporter genes during subclinical NE (11). This study further evaluated the effects of in ovo administration and water supplementation of a postbiotic on intestinal morphology, gut permeability, and the expression of several TJ proteins, stem cell markers, and immune response genes in broiler chickens during a subclinical NE challenge.

Histomorphological examination of intestinal segments is a widely used method for evaluating intestinal health, primarily through measurements of VH and CD (21). A greater VH and VH: CD ratio indicate an enhanced absorptive surface area relative to the proliferative crypt regions, suggesting enhanced nutrient absorption and overall intestinal function (22). Our results revealed that postbiotic supplementation decreased CD and increased the VH: CD ratio in the jejunum prior to the NE challenge. The intestinal tract of the NE-challenged control birds displayed apical necrosis, villus breakdown, and crypt hyperplasia at the peak challenge on d 21. In contrast, postbiotic supplementation mitigated these negative effects, preserving villus integrity and improving overall intestinal morphology. Similar results were observed in chicks infected with *C. perfringens*, where supplementation with *S. cerevisiae*-derived mannan oligosaccharides (MOS) improved intestinal morphology, humoral immune response, and gut microbiota metabolites (23). The beneficial effects of postbiotics on the development of intestinal villi and crypts may be attributed to bioactive metabolites, such as SCFAs, which are absorbed by enterocytes as a key energy source, or their potential indirect effect on the microbiota, collectively supporting intestinal barrier function (24). Therefore, early supplementation of postbiotics offers potential enhancement of intestinal morphology, possibly by promoting beneficial intestinal microbiota and modulating the immune response (25, 26).

The intestinal barrier is supported by mucin, forming a protective mucus layer on the intestinal lining, while TJ proteins, including claudins, OCLN, and ZO family members, act as seals between epithelial cells (27). The current study showed that the birds in ovo-injected with the postbiotic (PIW) had significantly greater mRNA abundance of CLDN1 and OCLN in the jejunum compared to the NC group on d 0. In ovo administration of postbiotics through the amnion during late-stage embryonic development may promote the colonization of beneficial gut bacteria after hatch, leading to improved intestinal integrity and nutrient digestibility (28). MUC2, produced by epithelial goblet cells, is an important glycoprotein in the chicken intestines that contributes to intestinal barrier function and homeostasis (29). The broilers supplemented with the postbiotic in ovo and in drinking water showed significantly greater MUC2 mRNA abundance in the ileum compared to the non-supplemented control birds on d 14. This finding aligns with a previous study reporting that broilers supplemented with a *Lactiplantibacillus plantarum*-based postbiotic exhibited greater MUC2 and OCLN expression in the jejunum on d 42 (30). Our study showed that the NE-challenged birds supplemented with the postbiotic through in ovo injection and drinking water displayed increased mRNA levels of ZO-1 in the jejunum on d 21. ZO-1 is present in intestinal epithelial cells and interacts with claudins and OCLN to enhance the stability of TJ proteins (31). Our results suggest that broilers supplemented with a postbiotic both in ovo and in drinking water have the potential to modulate intestinal integrity during an NE challenge.

Intestinal stem cells are located at the base of intestinal crypts, playing a vital role in tissue regeneration and repair, maintenance of intestinal integrity, and support of immune function. At hatch, in ovo supplementation of the postbiotic resulted in increased mRNA abundance of LGR5 in the ileum on d 0. LGR5 is involved in the Wnt signaling pathway that regulates stem cell growth and tissue renewal (32). This suggests that postbiotic supplementation during late embryogenesis promotes intestinal stem cell differentiation, ensures the proper function of the intestinal barrier, and supports efficient nutrient absorption. In the present study, the PIW birds exhibited higher mRNA abundance of OLFM4 in the jejunum and ileum on d 7 and d 14, respectively. These results coincide with the rapid growth of the intestine, as evidenced by increased VH and VH: CD ratio. Birds fed MOS derived from the cell wall of *S. cerevisiae* exhibited greater VH, thus enlarging the nutritive absorptive area and promoting cell proliferation in the intestinal mucosa (33). When the intestinal epithelial lining is damaged upon injury (e.g., during NE), enterocytes undergo apoptosis and are replaced by new cells generated by stem cells (34). The lower mRNA levels of LGR5 in the NE-challenged groups during peak infection are likely a consequence of a deregulated intestinal renewal process. This deregulation process during NE compromises the intestinal barrier, creating small gaps between TJ proteins that allow *C. perfringens* toxins to pass through and further exacerbating localized inflammation (35).

Several studies have reported that in ovo delivery of bioactives can significantly enhance gut development, promote maturation of the gastrointestinal tract-associated innate immune system, and improve overall post-hatch performance in chickens (36). Our study showed that the chicks supplemented with the postbiotic through in ovo injection and drinking water had significantly lower mRNA abundance of the proinflammatory cytokines TNF-α and IFN-γ compared to the NC group in the CT on d 14. In addition, lower mRNA levels of iNOS and IL-10 were observed in the CT of the postbiotic-treated groups on d 14. TNF-α and IFN-γ are involved in macrophage activation in chickens and act systematically to induce robust nitric oxide (NO) responses via the enzyme iNOS (37). These changes may result from the immunoregulatory effects of postbiotics, as shown by a previous study where in ovo supplementation with yeast cell wall-derived MOS and xylooligosaccharides reduced proinflammatory cytokines and enhanced immunity (38). Such modulation of immune signaling may facilitate the maturation and preservation of intestinal barrier integrity, which could explain the observed upregulation of ZO-1 and MUC2 expression in the present study (39). IL-10, an immunoregulatory cytokine that controls the immune response during inflammation by inhibiting pro-inflammatory cytokines such as IFN-γ, helps to counteract excessive inflammation (40). The lower mRNA abundance of pro-inflammatory cytokines and the immunoregulatory cytokine IL-10 in the postbiotic-treated group may indicate a well-regulated immune response and locally primed immunity prior to the NE challenge (37). This reduced expression could reflect a balanced immune system, likely poised to initiate a more rapid and coordinated immune response upon challenge.

Following this immune regulation, on d 21, the NE-challenged birds supplemented with the postbiotic displayed higher mRNA abundance of pro-inflammatory cytokines (e.g., TNF-α and iNOS) compared to the NE-challenged control group. This could be attributed to the enhanced Th1 immunomodulatory effects of certain postbiotic metabolites, such as MOS, SCFAs, antimicrobial peptides, polysaccharides, and proteins, or a combination of these components, which collectively help manage NE during peak infection (24, 41, 42). Similarly, chickens fed yeast-based β-glucans showed greater mRNA abundance of IL-10, IFN-γ, iNOS, and macrophage migration inhibitory factor in thymus tissues during an *Eimeria* challenge (43). The increased mRNA abundance of pro-inflammatory cytokines could be further supported by tissue repair, aligning with the elevated abundance of ZO-1 and improved intestinal morphology observed in the NE-challenged birds supplemented with the postbiotic. Postbiotics can modulate cytokine levels; however, discrepancies in findings have been observed due to variations in postbiotic composition, form, and inclusion levels, as well as environmental conditions and the presence or absence of a challenge (44). Further investigations are needed to better delineate the immune regulation associated with in ovo administration and water supplementation of postbiotics.

## 5 Conclusion

Supplementation of this postbiotic in ovo and in drinking water post-hatch directly influenced intestinal maturation and development, as demonstrated by a higher VH: CD ratio in the jejunum, increased mRNA abundance of TJ proteins and stem cell-related genes, and reduced mRNA levels of inflammatory cytokines prior to the NE challenge. Postbiotic supplementation in ovo and in drinking water provided protection against NE in the broiler chickens by significantly improving intestinal morphology, enhancing immune-related mRNA abundance, and strengthening the intestinal barrier.

## Data Availability

The original contributions presented in the study are included in the article/supplementary material, further inquiries can be directed to the corresponding author.

## References

[1] Emami NK Dalloul RA. Centennial review: recent developments in host-pathogen interactions during necrotic enteritis in poultry. Poult Sci. (2021) 100:101330. doi: 10.1016/J.PSJ.2021.101330, PMID: 34280643 PMC8318987

[2] Dalloul RA Lillehoj HS. Poultry coccidiosis: recent advancements in control measures and vaccine development. Expert Rev Vaccines. (2006) 5:143–63. doi: 10.1586/14760584.5.1.143, PMID: 16451116

[3] Prescott JF Parreira VR Mehdizadeh Gohari I Lepp D Gong J. The pathogenesis of necrotic enteritis in chickens: what we know and what we need to know: a review. Avian Pathol. (2016) 45:288–94. doi: 10.1080/03079457.2016.1139688, PMID: 26813023

[4] Salminen S Collado MC Endo A Hill C Lebeer S Quigley EMM. The international scientific Association of Probiotics and Prebiotics (ISAPP) consensus statement on the definition and scope of postbiotics. Nat Rev Gastroenterol Hepatol. (2021) 18:649–67. doi: 10.1038/s41575-021-00440-6, PMID: 33948025 PMC8387231

[5] Wegh CAM Geerlings SY Knol J Roeselers G Belzer C. Postbiotics and their potential applications in early life nutrition and beyond. Int J Mol Sci. (2019) 20:4673. doi: 10.3390/IJMS20194673, PMID: 31547172 PMC6801921

[6] Guo J Zhang Z Guan LL Yoon I Plaizier JC Khafipour E. Postbiotics from *Saccharomyces cerevisiae* fermentation stabilize microbiota in rumen liquid digesta during grain-based subacute ruminal acidosis (SARA) in lactating dairy cows. J Anim Sci Biotechnol. (2024) 15:101. doi: 10.1186/s40104-024-01056-x, PMID: 39085941 PMC11293205

[7] Chen J Wang Y You J Chen J Tian M Chen F. Effects of *Saccharomyces cerevisiae* fermentation product on the nutrient digestibility and ileal digesta characteristics of cannulated growing pigs fed corn- or barley-sorghum-based diets. Anim Feed Sci Technol. (2021) 274:114887. doi: 10.1016/j.anifeedsci.2021.114887

[8] Lensing M van der Klis JD Yoon I Moore DT. Efficacy of *Saccharomyces cerevisiae* fermentation product on intestinal health and productivity of coccidian-challenged laying hens. Poult Sci. (2012) 91:1590–7. doi: 10.3382/ps.2011-01508, PMID: 22700503

[9] Nelson JR Sobotik EB Athrey G Archer GS. Effects of supplementing yeast fermentate in the feed or drinking water on stress susceptibility, plasma chemistry, cytokine levels, antioxidant status, and stress- and immune-related gene expression of broiler chickens. Poult Sci. (2020) 99:3312–8. doi: 10.1016/j.psj.2020.03.037, PMID: 32616224 PMC7597835

[10] Ganda E Chakrabarti A Sardi MI Tench M Kozlowicz BK Norton SA. *Saccharomyces cerevisiae* fermentation product improves robustness of equine gut microbiome upon stress. Front Vet Sci. (2023) 10:1134092. doi: 10.3389/fvets.2023.1134092, PMID: 36908513 PMC9998945

[11] Dong B Calik A Blue CEC Dalloul RA. Impact of early postbiotic supplementation on broilers’ responses to subclinical necrotic enteritis. Poult Sci. (2024) 103:104420. doi: 10.1016/j.psj.2024.104420, PMID: 39454532 PMC11539447

[12] Nelson JR Archer GS. Effect of yeast fermentate supplementation on intestinal health and plasma biochemistry in heat-stressed Pekin ducks. Animals. (2019) 9:790. doi: 10.3390/ani9100790, PMID: 31614703 PMC6827150

[13] Gao W An K Li P Li L Xia Z. Dietary *Saccharomyces cerevisiae* improves intestinal flora structure and barrier function of Pekin ducks. Poult Sci. (2022) 102:101940. doi: 10.1016/j.psj.2022.101940, PMID: 36436368 PMC9700307

[14] Gao J Zhang HJ Wu SG Yu SH Yoon I Moore D. Effect of *Saccharomyces cerevisiae* fermentation product on immune functions of broilers challenged with Eimeria tenella. Poult Sci. (2009) 88:2141–51. doi: 10.3382/ps.2009-00151, PMID: 19762868

[15] Johnson CN Hashim MM Bailey CA Byrd JA Kogut MH Arsenault RJ. Feeding of yeast cell wall extracts during a necrotic enteritis challenge enhances cell growth, survival and immune signaling in the jejunum of broiler chickens. Poult Sci. (2020) 99:2955–66. doi: 10.1016/j.psj.2020.03.012, PMID: 32475430 PMC7597693

[16] Madej JP Bednarczyk M. Effect of in ovo-delivered prebiotics and synbiotics on the morphology and specific immune cell composition in the gut-associated lymphoid tissue. Poult Sci. (2016) 95:19–29. doi: 10.3382/ps/pev291, PMID: 26527705

[17] Oladokun S Adewole DI. In ovo delivery of bioactive substances: an alternative to the use of antibiotic growth promoters in poultry production—a review. J Appl Poult Res. (2020) 29:744–63. doi: 10.1016/j.japr.2020.06.002

[18] Barekatain R Chrystal PV Howarth GS McLaughlan CJ Gilani S Nattrass GS. Performance, intestinal permeability, and gene expression of selected tight junction proteins in broiler chickens fed reduced protein diets supplemented with arginine, glutamine, and glycine subjected to a leaky gut model. Poult Sci. (2019) 98:6761–71. doi: 10.3382/ps/pez393, PMID: 31328774 PMC6869755

[19] Livak KJ Schmittgen TD. Analysis of relative gene expression data using real-time quantitative PCR and the 2-ΔΔCT method. Methods. (2001) 25:402–8. doi: 10.1006/meth.2001.1262, PMID: 11846609

[20] Gharib-Naseri K Kheravii S Keerqin C Swick RA Choct M Wu S-B. Differential expression of intestinal genes in necrotic enteritis challenged broiler chickens with 2 different *Clostridium perfringens* strains. Poult Sci. (2021) 100:100886. doi: 10.1016/j.psj.2020.11.063, PMID: 33516477 PMC7936145

[21] Bindari YR Gerber PF. Centennial review: factors affecting the chicken gastrointestinal microbial composition and their association with gut health and productive performance. Poult Sci. (2021) 101:101612. doi: 10.1016/j.psj.2021.101612, PMID: 34872745 PMC8713025

[22] Lee J Goo D Sharma MK Ko H Shi H Paneru D. Effects of graded yeast cell wall supplementation on growth performance, immunity and intestinal development of broiler chickens raised in floor pens for 42 days. Poult Sci. (2025) 104:104695. doi: 10.1016/j.psj.2024.104695, PMID: 39721260 PMC11732452

[23] Ao Z Kocher A Choct M. Effects of dietary additives and early feeding on performance, gut development and immune status of broiler chickens challenged with *Clostridium perfringens*. Asian-Austral J Anim Sci. (2012) 25:541–51. doi: 10.5713/ajas.2011.11378, PMID: 25049595 PMC4092898

[24] Liu L Li Q Yang Y Guo A. Biological function of short-chain fatty acids and its regulation on intestinal health of poultry. Front Vet Sci. (2021) 8:736739. doi: 10.3389/fvets.2021.736739, PMID: 34733901 PMC8558227

[25] Sayed Y Hassan M Salem HM Al-Amry K Eid GE. Prophylactic influences of prebiotics on gut microbiome and immune response of heat-stressed broiler chickens. Sci Report. (2023) 13:13991–17. doi: 10.1038/s41598-023-40997-7, PMID: 37634024 PMC10460421

[26] Liu N Wang JQ Jia SC Chen YK Wang JP. Effect of yeast cell wall on the growth performance and gut health of broilers challenged with aflatoxin B1 and necrotic enteritis. Poult Sci. (2018) 97:477–84. doi: 10.3382/ps/pex342, PMID: 29211897

[27] Awad WA Hess C Hess M. Enteric pathogens and their toxin-induced disruption of the intestinal barrier through alteration of tight junctions in chickens. Toxins (Basel). (2017) 9:60. doi: 10.3390/toxins9020060, PMID: 28208612 PMC5331439

[28] Humam AM Loh TC Foo HL Izuddin WI Zulkifli I Samsudin AA. Supplementation of postbiotic RI11 improves antioxidant enzyme activity, upregulated gut barrier genes, and reduced cytokine, acute phase protein, and heat shock protein 70 gene expression levels in heat-stressed broilers. Poult Sci. (2021) 100:100908. doi: 10.1016/j.psj.2020.12.011, PMID: 33518339 PMC7936158

[29] Liu Y Yu Z Zhu L Ma S Luo Y Liang H. Orchestration of MUC2 — the key regulatory target of gut barrier and homeostasis: a review. Int J Biol Macromol. (2023) 236:123862. doi: 10.1016/j.ijbiomac.2023.123862, PMID: 36870625

[30] Chang HM Loh TC Foo HL Lim ETC. *Lactiplantibacillus plantarum* postbiotics: alternative of antibiotic growth promoter to ameliorate gut health in broiler chickens. Front Vet Sci. (2022) 9:883324. doi: 10.3389/fvets.2022.883324, PMID: 35859810 PMC9289564

[31] Bauer H Zweimueller-Mayer J Steinbacher P Lametschwandtner A Bauer HC. The dual role of zonula occludens (ZO) proteins. J Biomed Biotechnol. (2010) 2010:402593. doi: 10.1155/2010/402593, PMID: 20224657 PMC2836178

[32] Haegebarth A Clevers H. Wnt signaling, Lgr5, and stem cells in the intestine and skin. Am J Pathol. (2009) 174:715–21. doi: 10.2353/ajpath.2009.080758, PMID: 19197002 PMC2665733

[33] Oliveira MC Rodrigues EA Marques RH Gravena RA Guandolini GC Moraes VMB. Performance and morphology of intestinal mucosa of broilers fed mannan-oligosaccharides and enzymes. Arq Bras Med Vet Zootec. (2008) 60:442–8. doi: 10.1590/s0102-09352008000200025

[34] Emami NK Calik A White MB Young M Dalloul RA. Necrotic enteritis in broiler chickens: the role of tight junctions and mucosal immune responses in alleviating the effect of the disease. Microorganisms. (2019) 7:1–14. doi: 10.3390/microorganisms7080231, PMID: 31370350 PMC6723922

[35] Stewart AS Pratt-Phillips S Gonzalez LM. Alterations in intestinal permeability: the role of the “leaky gut” in health and disease. J Equine Vet Sci. (2017) 52:10–22. doi: 10.1016/j.jevs.2017.02.009, PMID: 31000910 PMC6467570

[36] Pender CM Kim S Potter TD Ritzi MM Young M Dalloul RA. In ovo supplementation of probiotics and its effects on performance and immune-related gene expression in broiler chicks. Poult Sci. (2017) 96:1052–62. doi: 10.3382/ps/pew381, PMID: 28158826

[37] Alizadeh M Shojadoost B Boodhoo N Raj S Sharif S. Molecular and cellular characterization of immunity conferred by *lactobacilli* against necrotic enteritis in chickens. Front Immunol. (2023) 14:1–13. doi: 10.3389/fimmu.2023.1301980, PMID: 38022592 PMC10662302

[38] Singh AK Tiwari UP Mishra B Jha R. Effects of in ovo delivered xylo- and mannan- oligosaccharides on growth performance, intestinal immunity, cecal short-chain fatty acids, and cecal microbiota of broilers. J Anim Sci Biotechnol. (2022) 13:13–6. doi: 10.1186/s40104-021-00666-z, PMID: 35130986 PMC8822640

[39] Luo Q Yang L Tumenjargal B Liu S Ma J Ning J. Effect of composite yeast culture on the jejunal barrier function, inflammatory response, and microbial community structure of laying hens during the late stage of egg production. Front Vet Sci. (2024) 11:1524726. doi: 10.3389/fvets.2024.1524726, PMID: 39744721 PMC11688808

[40] Moore KW De Waal MR Coffman RL O’Garra A. Interleukin-10 and the interleukin-10 receptor. Annu Rev Immunol. (2001) 19:683–765. doi: 10.1146/annurev.immunol.19.1.683, PMID: 11244051

[41] Yitbarek A Echeverry H Brady J Hernandez-Doria J Camelo-Jaimes G Sharif S. Innate immune response to yeast-derived carbohydrates in broiler chickens fed organic diets and challenged with *Clostridium perfringens*. Poult Sci. (2012) 91:1105–12. doi: 10.3382/ps.2011-02109, PMID: 22499867

[42] Saeed M Afzal Z Afzal F Khan RU Elnesr SS Alagawany M. Use of postbiotic as growth promoter in poultry industry: a review of current knowledge and future prospects. Food Sci Anim Resour. (2023) 43:1111–27. doi: 10.5851/kosfa.2023.e52, PMID: 37969321 PMC10636223

[43] Omara II Pender CM White MB Dalloul RA. The modulating effect of dietary beta-glucan supplementation on expression of immune response genes of broilers during a coccidiosis challenge. Animals. (2021) 11:1–12. doi: 10.3390/ani11010159, PMID: 33445562 PMC7827683

[44] Ahiwe EU Tedeschi Dos Santos TT Graham H Iji PA. Can probiotic or prebiotic yeast (*Saccharomyces cerevisiae*) serve as alternatives to in-feed antibiotics for healthy or disease-challenged broiler chickens?: a review. J Appl Poult Res. (2021) 30:100164. doi: 10.1016/j.japr.2021.100164

[45] Calik A Niraula A Dong B Blue CEC Fenster DA Dalloul RA. Iohexol-based assessment of intestinal permeability in broilers challenged with *Eimeria maxima*, *Clostridium perfringens* or both. Front Physiol. (2024) 15:1520346. doi: 10.3389/fphys.2024.1520346, PMID: 39759108 PMC11695284

[46] Li J Li J Jr Zhang SY Li RX Lin X Mi YL. Culture and characterization of chicken small intestinal crypts. Poult Sci. (2018) 97:1536–43. doi: 10.3382/ps/pey010, PMID: 29509914

[47] Emami NK Greene ES Kogut MH Dridi S. Heat stress and feed restriction distinctly affect performance, carcass and meat yield, intestinal integrity, and inflammatory (chemo) cytokines in broiler chickens. Front Physiol. (2021) 12:707757. doi: 10.3389/fphys.2021.707757, PMID: 34366895 PMC8339925

[48] Paul MS Paolucci S Barjesteh N Wood RD Schat KA Sharif S. Characterization of chicken thrombocyte responses to toll-like receptor ligands. PLoS One. (2012) 7:e43381. doi: 10.1371/journal.pone.0043381, PMID: 22916253 PMC3423363

